# Cortical specialisation to social stimuli from the first days to the second year of life: A rural Gambian cohort

**DOI:** 10.1016/j.dcn.2016.11.005

**Published:** 2016-11-27

**Authors:** S. Lloyd-Fox, K. Begus, D. Halliday, L. Pirazzoli, A. Blasi, M. Papademetriou, M.K. Darboe, A.M. Prentice, M.H. Johnson, S.E. Moore, C.E. Elwell

**Affiliations:** a Centre for Brain and Cognitive Development, Birkbeck, University of London, UK; b Department of Medical Physics and Biomedical Engineering, University College London, UK; c Cognitive Development Center, Central European University, Hungary; d Department of Psychology, University of Victoria, Canada; e MRC International Nutrition Group, MRC Unit, Gambia; f MRC Unit, Banjul, Gambia; g Division of Women’s Health, King’s College London, UK

**Keywords:** fNIRS, Infancy, Low- and middle-income countries, Nutrition, Poverty, Social cognition

## Abstract

Brain and nervous system development in human infants during the first 1000 days (conception to two years of age) is critical, and compromised development during this time (such as from under nutrition or poverty) can have life-long effects on physical growth and cognitive function. Cortical mapping of cognitive function during infancy is poorly understood in resource-poor settings due to the lack of transportable and low-cost neuroimaging methods. Having established a signature cortical response to social versus non-social visual and auditory stimuli in infants from 4 to 6 months of age in the UK, here we apply this functional Near Infrared Spectroscopy (fNIRS) paradigm to investigate social responses in infants from the first postnatal days to the second year of life in two contrasting environments: rural Gambian and urban UK. Results reveal robust, localized, socially selective brain responses from 9 to 24 months of life to both the visual and auditory stimuli. In contrast at 0–2 months of age infants exhibit non-social auditory selectivity, an effect that persists until 4–8 months when we observe a transition to greater social stimulus selectivity. These findings reveal a robust developmental curve of cortical specialisation over the first two years of life.

## 1 Introduction

Infants in resource-poor settings may be frequently exposed to a range of social, environmental, nutritional and pathological insults. Approximately 1 in 2 children are thought to live in poverty (Currie and Almond, 2011, UNICEF, 2013), and 165 million children worldwide are under nourished and stunted (UNICEF, 2013), the majority of whom live in Sub-Saharan Africa or South Asia. According to a recent study, one third of children in developing countries fail to reach their developmental milestones in cognitive and/or socio-emotional growth, with the largest number of affected children in sub- Saharan Africa (McCoy et al., 2016). This means that over 80 million children in low and middle income countries (LMICs) fail to develop a core set of age-appropriate skills that allow them to maintain attention, understand and follow simple directions, communicate and cooperate with others, control aggression, and solve complex problems. The absence of these skills has significant impact on their academic achievement and mental health into adulthood, and as such their potential to lead full and productive lives and support future generations. While many studies suggest that the presence of these risk factors in infancy has a lasting impact throughout the life course (Hackman and Farah, 2009, Martorell et al., 2010, Victora et al., 2008), almost nothing is known about the neural bases of these early deficits. The first 1000 days of life are a critical window for brain and nervous system maturation, and impaired development during this time can have a significant impact on cognitive outcome (Cusick and Georgieff, 2012, Mendez and Adair, 1999, Powell et al., 1995). To inform interventions that may reduce the impact of these insults, early detection of atypical neurocognitive function is required. However, to date there has been a lack of suitable methods for use from early infancy (Isaacs, 2013). Investigation of the developing brain in rural field settings has been broadly limited to behavioural assessments (Georgieff, 2007, Sabanathan et al., 2015). However, measurements of behaviour come with some limitations. Firstly, they can only be used to detect effects once they reach the point of observable behaviour, usually in the second year of life or later. For example, whilst behavioural measures have been unable to distinguish between infants with low and high-risk of developing autism (defined by a familial diagnosis) before the first year of life, several recent neuroimaging studies have identified differences in brain function in young infants (Elsabbagh et al., 2012, Elsabbagh et al., 2009, Fox et al., 2013, Guiraud et al., 2011, Lloyd-Fox et al., 2013, Luyster et al., 2011, McCleery et al., 2009). Furthermore, work on the relationship between family socio-economic status (SES) and infant brain development has evidenced atypical neural activity using electroencephalography (EEG) in six to nine month old infants from low SES backgrounds in the UK, highlighting the importance of the early-life environment on brain development (Tomalski et al., 2013). Secondly, there are issues relating to the implementation, cultural adaptation and standardisation of behavioural assessments between contrasting populations. For example, many standardised assessment measures are developed and normed within a limited number of high-income countries (Mullen, 1995). Therefore researchers need to develop country-specific norms for these measures or create independent measures and questionnaires for their own populations (i.e. Abubakar et al., 2016, Kariuki et al., 2016, Sabanathan et al., 2015). Adjustments to these measures can produce more robust and reliable datasets within populations, but can also hinder cross-cultural comparison due to issues with measurement equivalence.

Neuroimaging paradigms can be designed to be unbiased, objective and applicable across different populations. However, to date, there has been a lack of neuroimaging studies in infants in LMICs. In many instances this is because the engineering and technical issues associated with applying neuroimaging techniques in resource-poor settings have not been addressed. For example, the high cost and low portability of neuroimaging methods such as magnetic resonance imaging (MRI) has excluded their use in resource poor settings and field-based research. Direct measures of brain activity are possible in such settings using EEG methods, however their use can be limited by testing constraints (including the need for controlled temperature and humidity levels in hot countries, Kappenman and Luck, 2010), and most studies are not undertaken until infants reach the age of 18 months of age or older (Fernandes et al., 2014). Functional near infrared spectroscopy (fNIRS) is a non-invasive optical neuroimaging technique, which can measure cortical brain function. Infants wear lightweight headgear which facilitates the delivery to, and detection of near infrared light from the head. Changes in near infrared light intensity are a correlate of changes in haemodynamics and oxygenation arising from localized neuronal activity in the underlying cortical tissue (Villringer and Chance, 1997). fNIRS headgear can be rapidly administered and is well tolerated by young infants from birth. With optimal positioning of measurement channels (pairs of source lights and detectors), fNIRS generally allows for more specific spatial localization of activation with respect to EEG. Though the depth resolution of fNIRS is dependent on the age of the infant and the optical properties of the tissue (Fukui et al., 2003), and it offers lower spatial resolution relative to fMRI, it is similar in that it measures the haemodynamic response resulting from neuronal activation. Research from adults has shown a high degree of correlation between simultaneous recordings of haemodynamic responses with fNIRS and fMRI (Sato et al., 2013, Steinbrink et al., 2006). We believe fNIRS can be widely adopted for field based research due to its low cost (relative to other neuroimaging methods such as MRI), portability, ease of use with infants (Gervain et al., 2011, Lloyd-Fox et al., 2010) and clinical populations (Jackson and Kennedy, 2013, Kolyva et al., 2013), and suitability for use in naturalistic settings (Lloyd-Fox et al., 2015a).

**Fig. 1:**
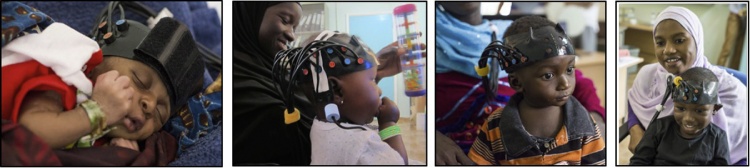
fNIRS headgear on a newborn (Cohort 1), 6 month old infant and 13 month old infant (Cohort 2: longitudinal) and a 2 year old toddler (Cohort 3).

In 2013 we transported an fNIRS neuroimaging system to a field station in rural Gambia and demonstrated its use to acquire maps of cortical function from young infants. The findings from our studies in 4–8 month Gambian infants were previously described by Lloyd-Fox et al. (Lloyd-Fox et al., 2014a). In this paper we present data from Gambian infants aged 19 days to 24 months of age (see Fig. 1).

The primary aim of the current work was to assess the specialisation of cortical activation in response to social cues from the first days of life to the second year. A secondary goal was to compare the responses from the rural Gambian cohorts with known responses in infants from an urban UK population. We use the term ‘social’ in this paper in the broadest sense, i.e. they are human-generated sensory stimuli in either the visual or auditory domain. This does not necessarily imply that these cues are intended to be communicative. We chose to use these stimuli for several reasons. Firstly, previous research suggests that infants are able to identify, and learn from, voices in their surroundings from a very early age (Ockleford et al., 1988), making this an ideal stimulus to use for our 0–24-month age range. Secondly, whilst there is an extensive and rich body of fNIRS research on infant language and speech processing (Quaresima et al., 2012), we chose to use non-speech vocalisations in the current paradigm because a suitable paradigm would have been challenging to develop for all of the languages spoken in the West Kiang district of The Gambia where Keneba is situated. We therefore felt that the non-speech vocalization paradigm would be more widely applicable across different languages and cultures. Thirdly, recent fNIRS research in typically developing infants from high-income urban environments has shown robust and consistent activation to social vs non-social visual and auditory stimuli in the inferior frontal, anterior temporal and posterior superior temporal – temporo parietal junction (pSTS-TPJ) regions of the cortex (Grossmann et al., 2010, Lloyd-Fox et al., 2012, Lloyd-Fox et al., 2009, Minagawa-Kawai et al., 2011). Therefore we were confident that we could optimize this paradigm across a wider age range. Having previously established this signature response in infants from 4 to 8 months of age in rural Gambia (Lloyd-Fox et al., 2014a), here we applied this paradigm to the investigation of social versus non-social cortical responses in infants from the first days of life to the second year.

## 2 Materials and methods

### 2.1 Participants

Participants were recruited from villages neighbouring a field station in Keneba, West Kiang District in The Gambia (see (Lloyd-Fox et al., 2014a) for further details), identified using the West Kiang Demographic Surveillance System (http://www.ing.mrc.ac.uk/research_areas/west_kiang_dss.aspx). All infants were born full term (37–42 weeks gestation) and with normal birth weight. A combination of prenatal growth retardation, poor-quality and often contaminated foods and high levels of infection cause moderate to severe growth faltering in height and weight gain from around 3 months of age in the local population (Lunn et al., 1991, Lunn, 2000, van der Merwe et al., 2013). Therefore exclusion criteria included weight-for-height or head circumference less than 3 z-scores below WHO standards. Growth measures for those included in the study indicate the infants were in the typical range for their age (see Table 1). Ethical approval was given by the joint Gambia Government − MRC Unit Ethics Committee, and written informed consent was obtained from all parents/carers prior to participation.

**Table 1:** Participant Characteristics.

Characteristics	0–2 mths	4–8 mths	9–13 mths	12–16 mths	18–24 mths
Sex (m/f)	14/4	10/14	15/10	9/10	7/9
Age (days)	41.0 ± 14.32^c^	174.4 ± 40.7	348.4 ± 37.8	428.2 ± 34.2	631.2 ± 76.0
Weight (kg)	4.59 ± 1.04	6.91 ± 0.75	8.12 ± 1.48	8.24 ± 0.84	9.42 ± 1.44
Length (cm)	54.9 ± 3.46	64.46 ± 3.39	70.4 ± 2.89	72.88 ± 2.8	79.87 ± 4.16
Head circumference H—C (cm)	37.5 ± 1.68	41.45 ± 1.3	43.85 ± 1.52	44.48 ± 1.30	45.91 ± 1.46
MUAC^a^ (cm)	12.3 ± 0.12	13.8 ± 0.59	14.1 ± 7.04	13.9 ± 0.55	14.1 ± 0.85

Growth anthropometric z-scores^b^					
Weight-for-age	−0.41 ± 1.2^d^	−0.75 ± 0.86	−1.28 ± 1.50	−1.49 ± 0.91	−1.51 ± 1.15
Length-for-age	−0.43 ± 1.08	−0.82 ± 1.21	−1.65 ± 1.08	−1.67 ± 0.98	−1.37 ± 1.04
H—C-for-age (HCZ)	−0.25 ± 0.9	−0.89 ± 0.99	−1.20 ± 1.16	−1.13 ± 0.93	−0.91 ± 0.88
Weight-for-length (WHZ)	−0.05 ± 1.1	−0.21 ±0.82	−0.57 ± 1.67	−1.67 ± 0.86	−1.16 ± 1.13

a Mid upper arm circumference.

b With the use of WHO reference curves.

c Mean ± SD (all such values).

d z score ± SD (all such values thereafter).

**Fig. 2:**
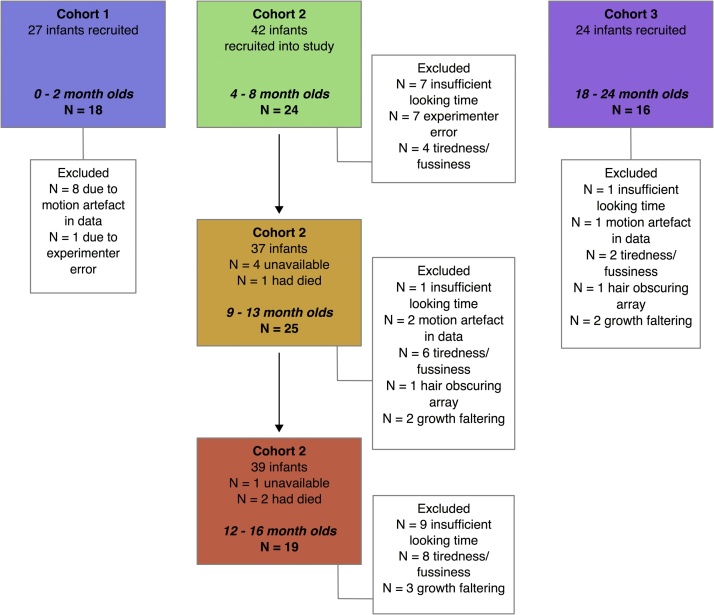
A flowchart illustrating the cohorts in the study.

These participants were recruited into one of three cohorts (see Fig. 2). Cohort 1 participated at *0–2 months* of age. Cohort 2, which included longitudinal data collection, participated at *4–8 months* of age (see previously published results in Lloyd-Fox et al., 2014a) and at two further sessions, six (aged *9–13 months*) and nine months (aged *12–16 months*) later. Cohort 3 participated at *18–24 months* of age.

Following each session, infant’s data could be excluded for the following reasons; due to (1) motion artefact in the data, (2) an insufficient number of valid trials according to looking time measures, (3) tiredness/fussiness resulting in session finishing early, (4) experimenter error, (5) hair obscuring array and preventing measurements, or (5) their weight for height (WHZ) or head circumference (HCZ) z-scores falling below −3. Therefore for Cohort 1, 18 infants participated in the study (4 female, mean age = 41.0 days, *SD* = 14.32): a further eight infants participated but were excluded from group analyses. For Cohort 2 (longitudinal), 42 infants were initially recruited into the study. At *4–8 months* of age (previously published in (Lloyd-Fox et al., 2014a)), 24 infants participated in this study (10 female, mean age = 174.4 days, *SD* = 40.7) and a further 18 infants participated but were excluded from group analyses. Six months later parents and infants were asked to return for a second session. Now aged *9–13 months*, 25 infants participated in this study (10 female, mean age = 348.4 days, *SD* = 37.8) and a further 12 infants participated but were excluded from further analyses. In addition five infants from the first session at *4–8 months* of age could not participate at this time point either because they were away from the region at time of testing (4 infants) or had died since the last visit (1 infant). Three months later parents and infants were asked to return for a final session. Now aged *12–16 months*, 19 infants participated in this study (10 female, mean age = 428.2 days, *SD* = 34.1) and a further 18 infants participated but were excluded from further data analyses. In addition three infants from the first session could not participate at this time point either because they were away from the region at time of testing (1 infant) or had died since the last visit (2 infants). For Cohort 3, 16 infants participated in the study (9 female, mean age = 631.2 days, *SD* = 76.0) and a further 8 infants participated but were excluded from the study. Fig. 2 provides detail of the participants tested in this study and reasons for exclusion.

### 2.2 Procedure

#### 2.2.1 fNIRS measurements

**Fig. 3:**
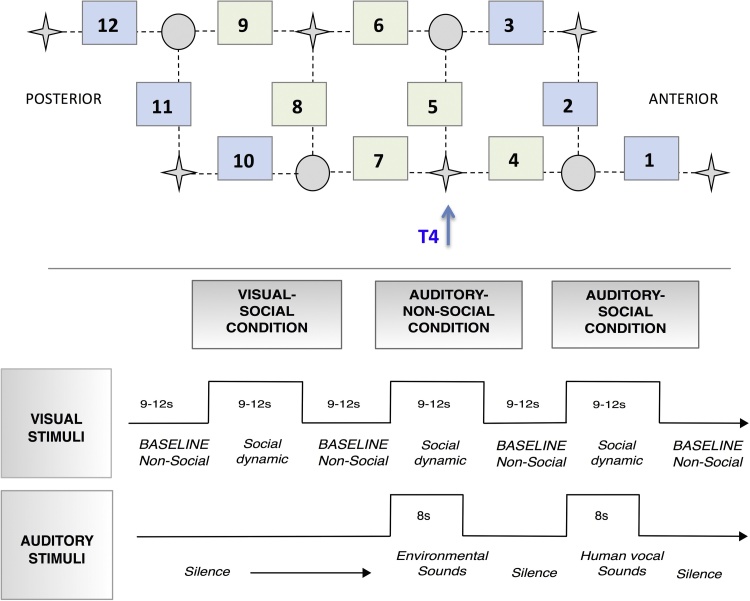
Upper panel – fNIRS headgear: **Cohort 2** at *4–8 and 12–16 months* of age wore the full array (blue and green channels), **Cohort 2** at *9–13 months* and **Cohort 3***(18–24 months)* wore the partial array (green). **Cohort 1** (*0–2 months*) wore an array covering channels 5, 6, 7, 8, 9, 10, 11 & 12. Source lights are indicated by a star and detectors by a circle. Lower panel – Stimulus presentation.

Infants wore custom-built fNIRS-CBCD headgear consisting of an array over the right hemisphere (Lloyd-Fox et al., 2010). Note that measurements were restricted to the right hemisphere as (1) our funding only allowed for a restricted number of sources and detectors with respect to the NIRS system used in the UK, and (2) we localized the channels to one hemisphere to ensure we could measure the entire temporal lobe area. This array varied in size and configuration according to the age group tested (see Fig. 3). The array contained up to a total of 12 channels (source-detector separations 2 cm), and infants were tested with the UCL optical topography system (Everdell et al., 2005). For **Cohort 1** we used a smaller array to adjust for the smaller head size at this age employing 8 channels to cover the same area of the head. For **Cohort 2** at *4–8 and 12–16 months* of age we used the full number of 12 channels. For **Cohort 2** at *9–13 months* of age and **Cohort 3** we employed a different shaped array to enable additional measurements over the prefrontal cortex (which was designed to test a different paradigm at the same visit when both of these age groups were tested). However this design resulted in sub optimal placement of channels over the posterior temporal ROI (pSTS-TPJ – posterior Superior Temporal Sulcus – Temporoparietal Junction), which meant that we may not have measured the full extent of the cortical activation for the visual social/non-social contrast at these age points (see Fig. 3). For the source detector separations used in this study, light transport models predict light penetration depths of up to approximately 1 cm from the skin surface, potentially allowing measurement of both the gyri and parts of the sulci near the surface of the cortex (Fukui et al., 2003, Richards et al., 2012, Salamon et al., 1990). With the use of the co-registration MRI-fNIRS data from our recent work (Lloyd-Fox et al., 2014b) we can approximate the underlying cortical anatomy of the fNIRS channels used in the current study. Therefore, we are confident that we can localize our investigation to specific regions of the social brain network and draw comparisons with findings from adult populations. Before the infants began the study, head measurements were taken to align the headgear with 10–20 coordinates (Lloyd-Fox et al., 2014b). The head measurements showed that the infants’ head circumference did not change considerably between 4 and 24 months of age so we utilized the same 2 cm separations throughout the cohorts. The headgear was placed with the source light optode positioned between channel 4 and 7 on Fig. 3 centred above the pre-auricular point (T4 according to the 10–20 system).

The protocol for **Cohorts 2 and 3** followed an identical procedure to that outlined in our previously published study (Lloyd-Fox et al., 2014a). For the readers convenience we repeat text from this previous work, but with additional information provided about the different testing protocol followed for **Cohort 1**. Once the fNIRS headgear was placed on their heads, the infants sat on their parent’s lap in front of a screen (for **Cohorts 2 & 3**) or asleep on a mattress or their parent’s lap for **Cohort 1**. For **Cohort 1** we waited for the swaddled infants to fall asleep, then wrapped the headband around their head and waited for them to settle before beginning the study. We prioritized placing the infants on a cushioned mattress for the study but if they were restless they were held in their parent’s arms, therefore whilst sleep state was prioritized some infants were tested while in a quiet alert state. For two of the infants they became alert and then fussy and it was necessary to feed them during the study and trials during this section of the data were excluded from data analysis. For all participants the parent was instructed to refrain from interacting with the infant during the stimuli presentation unless the infant became fussy or sought their attention. The sequence of stimulus presentation has been used in previous research (Lloyd-Fox et al., 2012, Lloyd-Fox et al., 2013) and is illustrated in Fig. 3. The conditions alternated one after the other, with a period of baseline between each. The three types of conditions (*visual-social (silent), auditory social*, *auditory non-social)* were presented in the same order across infants in a repeating loop (V-S, A-NS, A-S, V-S, A-S, A-NS) of trials (single presentation of a condition) until the infants became bored or fussy as judged by the experimenter who was monitoring their behaviour. A restriction of studying auditory processing in awake infants of this age is that they need to be presented with concurrent visual stimulation to reduce infant movement and thus artefact in the signal. We chose to employ the same visual stimuli during the presentation of the auditory stimuli that we collected data from when auditory stimulation was absent. For **Cohort 1** we used the same paradigm and equipment, placing the TV monitor at the same distance from the infants’ head as used for **Cohorts 2 & 3**.

### 2.3 Visual stimuli

These consisted of full-color, life-size (head and shoulders only) social videos of adults (Gambian nationals) who either moved their eyes left or right or performed hand games —“Peek-a-boo” and “Incy-Wincy Spider.” Two visual social videos were presented for varying duration over each 9–12 s trial to avoid inducing anticipatory brain activity. To ensure infants’ continuous attention – especially since the social visual stimuli was also presented during auditory trials – there were six different visual social videos (two actors; three types of social video), while each auditory condition employed two different recordings (two speakers; one recording each – see below). During the baseline, visual stimuli were displayed, which consisted of full-color still images of different types of transport (i.e., cars and helicopters) presented randomly for a pseudorandom duration (1–3 s) for 9–12 s (Lloyd-Fox et al., 2012). Dynamic non-social baseline stimuli have also been used in previous work investigating responses to visual social dynamic stimuli, and have been found to produce similar effects to the static non-social baseline used in the current study (Lloyd-Fox et al., 2011, Lloyd-Fox et al., 2009). These visual stimuli were displayed on a 24-inch plasma screen with a viewing distance of approximately 100 cm.

### 2.4 Auditory stimuli

During the presentation of visual stimuli the infants were presented with auditory stimuli (see Fig. 3) at the onset of two out of every three of the trials. The content and duration of the social videos (9–12 s) were not synchronized with the auditory stimuli. Each auditory stimulus presentation lasted 8 s and consisted of four different sounds (of vocal or non-vocal stimuli) presented for 0.37–2.92 s each, interleaved by short silence periods (of 0.16–0.24 s). The two auditory conditions were equivalent in terms of average sound intensity and duration (p = 0.65). Within the auditory social condition infants were presented with non- speech adult vocalizations of two speakers (who coughed, yawned, laughed, and cried). Within the auditory non-social condition, the infants were presented with common environmental sounds (that were not human or animal produced, but were likely to be familiar to infants of this age; running water, bells and rattles). Vocal and non- vocal stimuli were chosen from the Montreal Affective Voices (for more detail, see (Belin et al., 2000)) and the stimuli of the voice functional localizer (http://vnl.psy.gla.ac.uk/resources_main.php). Additional non-vocal stimuli (toy sounds) were also recorded by the authors (Blasi et al., 2011).

### 2.5 Data processing and analysis

The NIRS system measured the light attenuation from each source detector pair. These light attenuation measures were used to calculate changes in the concentration of oxy-haemoglobin (HbO_2_) and deoxy-haemoglobin (HHb) in μMol which were used as haemodynamic indicators of cortical neural activity (Obrig and Villringer, 2003). The analysis procedure followed a similar protocol to previous infant research. Initially, the recorded near infrared attenuation measurements for each infant were analyzed separately. Trials were rejected from further analysis based on looking time measures (coded offline by a researcher unfamiliar with the study’s aims, trials with >60% trial looking were considered valid) and channels were rejected based on the quality of the signal, using artefact detection algorithms (Lloyd-Fox et al., 2010, Lloyd-Fox et al., 2009). For each infant, the light attenuation signal was low-pass filtered, using a cut off frequency of 1.8 Hz. The data was then divided into blocks consisting of 4 s of the baseline trial prior to the onset of the stimulus, the experimental stimulus trial, plus the following baseline. The light attenuation data was detrended with a linear fit between the first and last 4 s of each block. The data were then converted into changes in concentration (μMol) in HbO_2_ and HHb using the modified Beer Lambert law (Delpy et al., 1988) and assuming a differential pathlength factor for infants (5.13; based on (Duncan et al., 1995)). A minimum of three valid trials per condition was set as a threshold for inclusion within infants. Inclusion criteria required each channel to contain valid data in all three experimental conditions. For each infant, the trials and channels that survived these rejection criteria were entered into further analyses. Following this, valid trials for each condition (*visual-social (silent), auditory social*, *auditory non-social)* were averaged together within channels for each infant, and a time course of the mean concentration change in HbO_2_ and HHb was compiled for each channel. Two time windows were selected between 8–12 and 12–16 s post-stimulus onset for each trial. This period of time was selected to include the range of maximum concentration changes observed across infants for HbO_2_ and HHb, based on visual inspection of the current data, and informed by data analysis approaches using the same paradigm in previous cohorts (Lloyd-Fox et al., 2013, Lloyd-Fox et al., 2012, Lloyd-Fox et al., 2009). The time window was split into two epochs to allow us to investigate latency further, as we have noted differences in the timing and shape of the response across the different visual and auditory conditions in previous research. For each channel, statistical comparisons (two-tailed *t*-tests) of the maximum change (amplitude) (in HbO_2_ (increase in chromophore concentration) and/or HHb (decrease in chromophore concentration)) were performed for the (1) *visual-social (silent)* condition compared to the non-social baseline condition *(with silence)* and the (2) direct comparison of the *auditory social* and *non-social* conditions during the specified time windows. In addition for **Cohort 1** only, the contrast between the two auditory conditions and silence was also performed to explore the age dependent specialisation of this response further. Either a significant increase in HbO_2_ concentration, or a significant decrease in HHb, is commonly accepted as an indicator of cortical activation in infant work, however in accordance with previous research (Lloyd-Fox et al., 2010) we found that the majority of the significant effects were in HbO_2_ and so focused our results on this signal. The significant HHb responses are reported in Supplementary Data. To resolve statistical problems of multiple comparisons for these group analyses we applied the false discovery rate (FDR) correction (Benjamini and Hochberg, 1995). The channels that did not survive this correction are highlighted in Table 2, however we chose to report the results in full in the Results section as the replication of effect across ages allays the need for a strict FDR correction.

**Table 2:** Significant HbO_2_ responses to the Social > Non-Social Visual and Auditory contrasts across the three Cohorts.

4–8 months (Cohort 2)										12 − 16 months (Cohort 2)									
	Visual Social > Non-Social					Auditory Social > Non-Social					Visual Social > Non-Social					Auditory Social > Non-Social			
Ch	*TW*	*t*	*p*	*df*	Ch	*TW*	*t*	*p*	*df*	Ch	*TW*	*t*	*p*	*df*	Ch	*TW*	*t*	*p*	*df*
8 *	12–16	2.55	0.018	*23*						6*	12–16	2.22	0.04	*18*	5 *	12–16	2.83	0.011	*18*
**9**	12–16	3.57	0.0016	*23*						**9**	8–12	3.63	0.002	*17*	6 *	12–16	2.16	0.045	*18*
11 *	8–12	2.56	0.018	*23*						**9**	12–16	3.55	0.0025	*17*					
**11**	12–16	3.54	0.0017	*23*						**11**	8–12	3.67	0.0018	*17*					
**12**	8–12	3.21	0.0004	*23*						**11**	12–16	3.39	0.0035	*17*					
	12–16	5.15	0.00003	*23*						**12**	8–12	4.63	0.0002	*17*					
										**12**	12–16	4.60	0.0003	*17*					

	9–13 months (Cohort 2)									18–24 months (Cohort 3)									
	Visual Social > Non-Social					Auditory Social > Non-Social					Visual Social > Non-Social					Auditory Social > Non-Social			
**Ch**	*TW*	*t*	*p*	*df*	**Ch**	*TW*	*t*	*p*	*df*	**Ch**	*TW*	*t*	*p*	*df*	**Ch**	*TW*	*t*	*p*	*df*
9 *	12–16	2.35	0.027	*24*	5 *	12–16	2.74	0.011	*24*	9 *	12–16	2.21	0.045	*13*	**5**	12–16	4.11	0.0009	*15*

0–2 months (Cohort 1)										0–2 months (Cohort 1)				
	Auditory Social > Silence					Auditory Non-Social > Silence					Auditory Non-Social > Non-Social			
**Ch**	*TW*	*t*	*p*	*df*	**Ch**	*TW*	*t*	*p*	*df*	**Ch**	*TW*	*t*	*p*	*df*
1 *	8–12	2.46	0.025	*17*	**1**	8–12	4.90	0.00016	*17*	6 *	8–12	2.45	0.025	*17*
1 *	12–16	2.37	0.03	*17*	**4**	8–12	4.23	0.0006	*17*					
2 *	8–12	2.35	0.032	*17*	4 *	12–16	3.30	0.0043	*17*					
2 *	12–16	2.39	0.029	*17*	**5**	8–12	4.77	0.00021	*17*					
**4**	8–12	3.29	0.0044	*17*	**6**	8–12	3.38	0.0036	*17*					
	12–16	4.57	0.0003	*17*	6 *	12–16	2.47	0.025	*17*					
					8 *	8–12	2.14	0.047	*17*					

## 3 Results

The findings reveal localized patterns of activation in regions of the posterior superior temporal, anterior temporal and inferior frontal cortex to the visual and auditory social stimuli, in concordance with previous cohorts of infants studied in the UK.

### 3.1 Visual social versus non-social

**Fig. 4:**
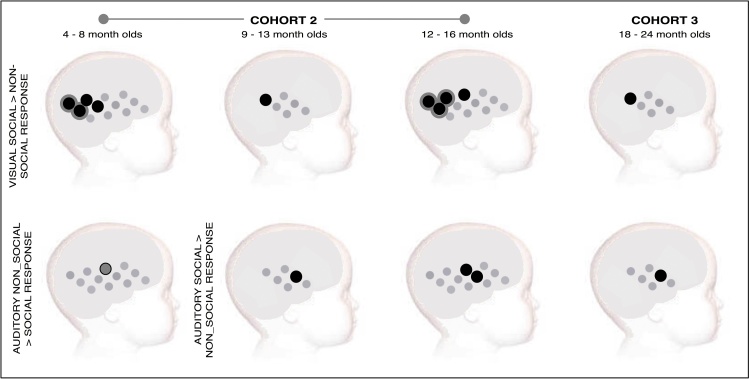
Significant HbO_2_ haemodynamic responses for each age group in the visual social > non-social contrast and the auditory social versus non-social contrasts. Note that for the auditory contrast for **Cohort 2** when the infants are aged *4–8 months* they evidence a significant non-social > social response, whilst by the next session when they are aged *9–13 months* and beyond, they show a social > non-social response (similar to **Cohort 3** at *18–24 months* of age). No auditory non-social > social responses were found between 9 and 24 months of age. Significant results are shown for two time windows, 8–12 s post stimulus onset (grey) and 12–16 s post stimulus onset (black).

**Fig. 5:**
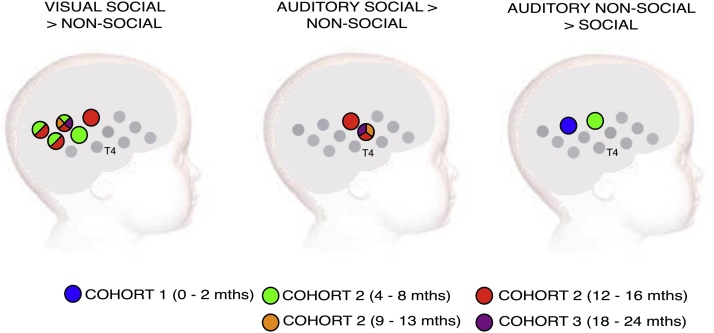
A summary of the locations of the significant HbO_2_ haemodynamic responses for each age group in the visual and auditory social versus non-social contrasts. This figure combines all significant results across the time windows 8 − 12 and 12–16 s post stimulus onset. Note that for **Cohort 2** at 4–8 months of age the infants do exhibit a significant auditory social > non-social response, but only in HHb and is therefore not illustrated here (see Supplementary Table 2 for details of significant HHb responses).

To assess the responses to the visual social stimuli the *visual-social (silent)* condition was analysed relative to the non-social baseline condition *(with silence)* (*t*-test, two-tailed) for each cohort (at each age point: see Table 2) in two time epochs (1) 8–12 s and (2) 12–16 s post stimulus onset. Note that **Cohort 1** did not contribute data to this contrast as they were either asleep or in a position where they were unable to view the visual stimuli. This analysis revealed significant increases in HbO_2_ centered over the posterior area of the arrays (see Fig. 4, Fig. 5), corresponding to the posterior STS-TPJ region of the cortex.

**Fig. 6:**
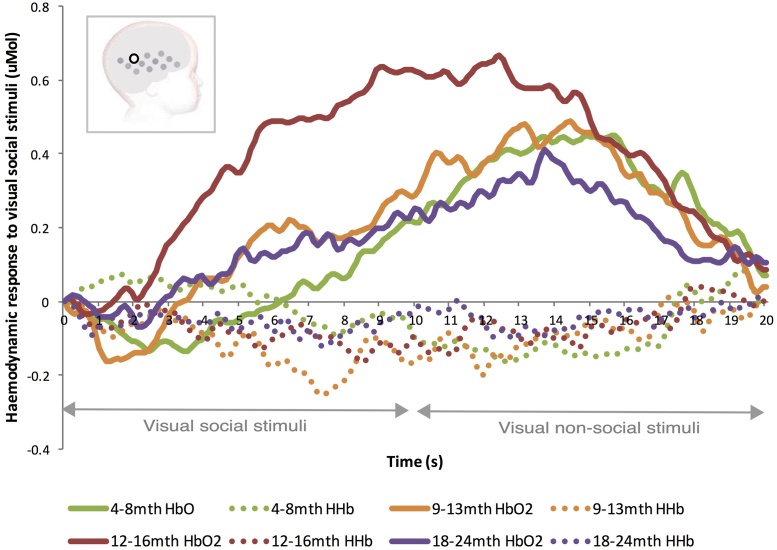
Haemodynamic time courses of the response to the visual social stimuli in Channel 9 for **Cohort 2** (longitudinal) at *4–8 months* (green), *9–13 months* (orange) and *12–16 months* (red) and for **Cohort 3** (*18–24 months)* (purple). Note HbO_2_ responses are in full, while HHb responses are dashed, and the location of this response is indicated on the schematic of the head.

As shown in Fig. 5 the *visual-social* *>* *non-social* response was localized to the same region from 4 to 24 months of age with one channel (Channel 9) consistently revealing significant activation across all four cohorts. Haemodynamic time courses for Channel 9 are shown in Fig. 6. Responses at *9–13 and 18–24 months* were less robust than at *4–8 and 12–16 months* of age due to the sub-optimal headgear placement (this may have contributed to the diminished response seen in the time course of **Cohort 3** *(18–24 months)* in Fig. 5 as coverage may not have reached the area of peak activation for this age group). No significant HHb responses were found for either the 8–12 or 12–16 s time epochs of analysis.

### 3.2 Auditory social versus non-Social

**Fig. 7:**
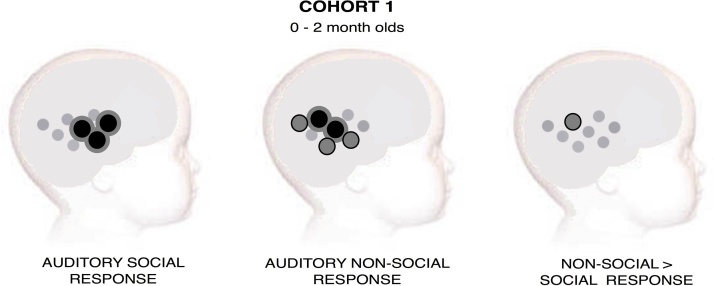
Significant HbO_2_ haemodynamic responses for **Cohort 1** (*0–2 month* olds): (1) *auditory social* vs silence, (2) *non-social* vs silence and (3) *non-social* *>* *social* contrasts. Significant results are shown for two time windows, 8–12 s post stimulus onset (grey) and 12–16 s post stimulus onset (black).

For **Cohort 1** initial statistical analyses of *auditory social* and *non-social* responses compared with silence (visual social only) were conducted (see Fig. 7) for each time epoch (8–12 and 12–16 s post stimulus onset). This analysis revealed a more widespread HbO_2_ response to the non-social stimuli (5 significant channels) compared with the social stimuli (3 significant channels). One of these channels also revealed a significant HHb response.

**Fig. 8:**
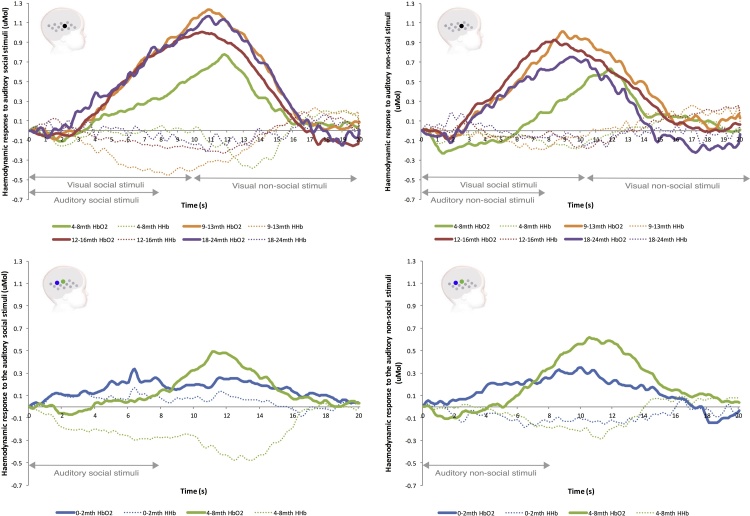
Haemodynamic time courses of the response to the auditory social stimuli (left panels) and non-social stimuli (right panels) in a socially selective channel (on upper row) for **Cohort 2** (longitudinal) at *4–8 months* (green), *9–13 months* (orange) and *12–16 months* (red) and for **Cohort 3** (*18–24 months)* (purple) and two non-socially selective channels (on lower row) for **Cohort 1** (*0–2 months)* (blue) and **Cohort 2** at *4–8 months* (green). Note HbO_2_ responses are in full, while HHb responses are dashed, and the location of this response is indicated on the schematics of the head (9–24 month olds (black), 0–2 month olds (blue) and 4–8 month olds (green).

Following this, paired sample channel-by-channel *t*-tests (two-tailed) were performed to assess the presence of auditory social and non-social selective activation across each cohort (see Table 2). An age dependent response was revealed across 0–24 months of age. For **Cohort 1** *(0–2 months)* and **Cohort 2** at *4–8 months* significant *non-social* *>* *social* HbO_2_ selectivity was evidenced in a posterior temporal region of the array (see Figs. 4, 5 and 7). As reported previously (Lloyd-Fox et al., 2014a), a *social* *>* *non-social response* was also evident in the 4–8 month old infants but confined to HHb (the analyses of HHb with the new time epochs are reported in Supplementary Data and confirm this previous finding). For **Cohort 2** at *9–13 and 12–16 months* of age, and for **Cohort 3** (*18–24 months*) significant *social* *>* *non-social* HbO_2_ selectivity was evidenced in an anterior temporal region of the array (see Fig. 4 and 5). These significant responses were localized within the same 3 channels across 9–24 months of age (see Fig. 5 & 6). There were no significant HHb responses in **Cohort 2** at *9–13 and 12–16 months* of age, and **Cohort 3** (*18–24 months*). Haemodynamic time courses for the channels showing significant social selectivity in **Cohort 2** at *9–13 and 12–16 months* of age, and **Cohort 3** (*18–24 months*) and non-social selectivity for **Cohort 1** *(0–2 months)* and **Cohort 2** at *4–8 months* are shown in Fig. 8.

## 4 Discussion

We have successfully implemented a social versus non-social fNIRS paradigm in two contrasting environments: rural Gambian and urban UK. To our knowledge this research in The Gambia is the first neuroimaging study to investigate cortical specialisation to social cues across such a wide span of early development, with participants ranging in age from newborn to toddlerhood. Furthermore, the stability of the elicited social response from 9 to 24 months, and the transition from non-social to social selectivity seen prior to this age, provides us with a robust developmental curve of specialisation to social cues over the first two years of life.

With the use of fNIRS we were able to measure localized brain responses to visual and auditory social cues across the first two years of life. For the visual social versus non-social contrast, the response was remarkably consistent across 4–24 months of age. In the two measurement channels that were placed in the same position over all four time points (**Cohort 2** at 4–8, 9–13, and 12–16 months of age and **Cohort 3** at 18–24 months of age), a significant response was found in the same channel at each age. Furthermore, for the longitudinal cohort the response appears to become more rapid with age, with a faster rise to peak seen at each subsequent age point. Using co-registration fNIRS – MRI data from previous cohorts of UK infants we were able to extrapolate the approximate position of these channels over underlying anatomy (Lloyd-Fox et al., 2014b, Lloyd-Fox et al., 2015b) and identify that this region of the array was positioned over the pSTS-TPJ. These responses largely replicated our previous findings from the UK in *4–8 month* old infants (Lloyd-Fox et al., 2013, Lloyd-Fox et al., 2012, Lloyd-Fox et al., 2009). Furthermore, given the interest in comparing the results from the Gambian cohorts with age-matched data in other participants, using an identical protocol we were able to collect data from a cohort of *12–16 month* olds in the UK (see Box 1). Despite the smaller sample size (N = 12), we found very similar results in this cohort with significant responses localized within the same region of pSTS-TPJ channels, supporting the current findings.

Box 1 **xxx.**

Alt-text: Box 1

The auditory social versus non-social contrast, which was used at all five age points from 0 to 24 months of age, revealed evidence of a developmental change in specialisation for auditory social stimuli across infancy. Whilst the 0–2 (**Cohort 1**) and 4–8 (**Cohort 2**) month olds evidenced significant HbO_2_ auditory non-social selectivity (non-social > social) in a region of channels localized over the pSTS-TPJ, **Cohort 2** at 9–13 and 12–16 months of age and the 18–24 month olds (**Cohort 3**) evidenced significant HbO_2_ auditory social selectivity (social > non-social) in a region of channels localized over the anterior temporal cortex. Furthermore, the group averaged haemodynamic responses of the infants from 9 to 24 months of age to the social and non-social auditory stimuli are remarkably similar, despite the contributed data being derived from different combinations of infants at each age point (within the longitudinal cohort it was dependent on those available for follow up, and who then contributed valid data, and Cohort 3 compromised a cross-sectional sample). In contrast the averaged haemodynamic responses of the 4–8 month olds (Cohort 2) suggest that the lack of social selectivity and evident non-social selectivity may be due to a delayed response to the social stimuli. The non-social selectivity is only apparent during the earlier time epoch and the response to both stimuli becomes equivalent at a later time point during stimulus presentation. Although speech perception in newborn infants is well described (Dehaene-Lambertz and Spelke, 2015, Gervain et al., 2008, Pena et al., 2003, Vannasing et al., 2016), responses to human vocalisations are less well known. To allow us to compare these responses with a UK cohort, in collaboration with colleagues in Cambridge, we were able to use the auditory paradigm with newborn infants at the local maternity hospital. The findings from the UK cohort of 1–4 day old newborns were consistent with the current findings (Chuen Wai Lee, Topun Austin et al. unpublished results). Widespread non-social responses (non-social > silence) were found, whilst an isolated region of the anterior temporal lobe responded to social sounds (social > silence). Furthermore a significant non-social > social selective response was localized within the pSTS-TPJ region of the cortex in these newborns. These findings are also in line with other recent research (Cristia et al., 2014) suggesting auditory responses to such cues are less specialized at this age. This comparison with previous research at 0–2 months is also important, as whilst in these other studies all infants were asleep, in the current study some of our participants in **Cohort 1** were in a quiet alert state. Therefore it is important to see that the responses were largely replicated across the Gambian and UK infants despite these differences in their state of alertness at time of test. Previous research with 4–7 month olds would suggest that auditory social > non-social selectivity emerges from the first months of life becoming more robust in the second half of the first year of life (Grossmann et al., 2010, Lloyd-Fox et al., 2012). Grossmann and colleagues only found social selectivity once the infants reached 7 months of age, and Lloyd-Fox and colleagues reported the strength of the response across 4–7 months of age correlated with age of infant. In the current work, we did find evidence of a social > non-social response at this age point but only within the HHb response. Interestingly, this was also the case for one of our previous studies in UK infants (Lloyd-Fox et al., 2012), whilst in other infants we have found a significant HbO_2_ selective response (Lloyd-Fox et al., 2013). Collectively, this research supports the view that this age range marks a shift in specialisation to social over non-social sounds and future research should focus on both HbO_2_ and HHb responses in this age group. In contrast, by 9 months of age onwards the social > non-social selectivity (and absence of non-social > social selectivity) becomes a robust response localized within a specific anterior temporal region of the cortex with all three of the older aged cohorts showing responses in this area. Furthermore, in support of these findings, the location and selectivity of this response was replicated in our UK 12–16-month-old cohort (see Box 1).

There are a number of potential challenges in performing neuroimaging studies in a rural location in The Gambia, including transportation of the instrumentation, and introduction of experimental methods to a new community and population of Africa infants. The portability of the fNIRS technology allowed us to transport it to the village of Keneba with relative ease. The technology and experimental paradigms were readily accepted by infants, parents and local field staff. For many of these infants this may have been the first time that they have viewed a TV monitor, and so we should be conscious of this in our interpretation of the results, nevertheless the responses in the Gambian infants were remarkably consistent with the UK population and infants were calm and attentive during the study. Indeed, the attrition rates were within the standard range for infant fNIRS studies with approximately 20–30% excluded for inattention or fussiness using a three condition contrast design (previous research indicates that in awake infants attrition can increase by approximately 10% with each additional experimental condition employed – see review by (Lloyd-Fox et al., 2010)). For the first visit of Cohort 2 (4–8 months) 7 out of the 42 infants were also excluded due to experimenter error. However this is expected considering that this data was collected at the first session of fNIRS testing in the Gambia, and due to time and budget constraints, we began the study without any lead in time to train with new staff and in the new setting. The infants excluded due to motion artifact or experimenter error in Cohort 1 (0–2 months) were also mostly those infants tested at the beginning when we were getting used to conducting fNIRS studies in this age group (as this was the first time the team had worked with such young infants) and so the infant position and study setup for data collection was optimized over time. During later visits there was no attrition due to this factor. For Cohort 2 at 12–16 months of age infants, they seem to have been more liable to look away or fuss out compared to the other age points, however this is behaviour consistently reported at this age point in developmental research. Furthermore, children who were excluded due to fussiness or inattention were not more likely to be those with very low HCZ or WHZ (only 3 infants under this exclusion category also had z scores under – 3) indicating that these infants can be measured with fNIRS. Other than growth faltering, we used the same exclusion criteria in The Gambia as we would have in the UK, and so as long as they were well enough to contribute enough valid data for a session, they were included in the study. Specific tests for ongoing infections or neurodevelopmental problems were not administered on the day of the visit and future larger scale research projects should take this into account. The CBCD designed headgear fit well on each age of infant and provided robust signals. It may be pertinent to note here that the restriction on funding that allowed us to only measure the right hemisphere may have been an advantage as it allowed for optimal fit of the headgear over the region of interest with a headband less liable to move on the head and cause artefact in the data. For example, once the infants’ hair became thicker at older age points we sometimes had difficulty measuring through braids on the female participants but found we could measure robust signals between the braids on the same participant. Balanced against this advantage of a smaller headgear providing more robust signals, was the issue of trying to run more than one cognitive paradigm with a single optical array design. The limited number of channels in the optical arrays only allowed us to measure a certain number of cortical regions. Therefore for one testing time point (when we were following up our longitudinal cohort at 9–13 month olds and testing 18–24 month olds) in Keneba we compromised data collection on the social paradigm because we only had two channels placed over our ROI for the visual social response rather than the original four channels. In particular, this may have contributed to the diminished response seen in the time course at 18–24 months in Fig. 5. Whilst the pSTS-TPJ ROI was within reach in the 9–13 months who wore this headgear design, coverage in this oldest age group may not have reached the area of peak activation. Though we had checked that head circumference did not change significantly between 9 and 24 months of age in these Cohorts, and therefore utilised the same sized headband, it may be that the pSTS-TPJ had extended out of reach of some of our measurement channels in a ventro-dorsal, rather than an anterior-posterior direction, by 18–24 months (Kabdebon et al., 2014, Lloyd-Fox et al., 2014b). In retrospect, and with information now available from this more recent research, we can see that future headgear designs should be more sensitive to these changes in anatomy over this wider age range by employing a larger number of channels over regions of interest to account for individual variability in anatomy.

We believe that these experiences have allowed us to optimize data collection across 0–24 months of age for research in field settings such as Keneba and this knowledge can be, and indeed is being, used to establish fNIRS research in other similar settings and for larger scale studies. To optimize higher rates of valid data we would recommend (1) a brief period of training with new staff alongside an experienced researcher while the team learns how to successfully use fNIRS with infants/children; (2) headgear which is stable, optimized for brain regions of interest, and able to measure through hair (our 60-channel bilateral headgear is currently being successfully used with 36 month olds in Bangladesh); (3) acknowledgement that data attrition will likely be higher with certain age ranges (i.e. 12–16 months of age); (4) optimized paradigms for successful data collection (i.e. a 2 condition contrast design would result in <20% data dropout, and we have previously conducted a 1 condition design which had an attrition rate of 4% − Lloyd-Fox et al., 2009); and (5) ideally more than one fNIRS session per individual and larger sample sizes to allow one to trace developmental change, while accounting for attrition of data. With these factors taken into account we are confident that fNIRS can become a valid measure for larger scale research studies.

We have now demonstrated that fNIRS can be easily implemented in a resource-poor rural setting and used from the first few days of life through to toddlerhood to provide quantitative and objective markers of neurocognitive function. Furthermore, the stability of the elicited social response from 9 to 24 months, and the transition from non-social to social selectivity seen prior to this age, provides us with a robust developmental curve of specialisation to social cues over the first two years of life. An area that merits future investigation is to further interrogate the specifics of the neurovascular response by using a mathematical model of cerebral blood flow and metabolism (Banaji et al., 2008) to more fully interpret the hemodynamic responses across our five age points. Given the stability of this developmental curve of specialisation, future research of compromised development could use this fNIRS paradigm to interrogate atypicalities in brain function and their association with risk factors such as under nutrition and poverty. For example, in infants tested with fNIRS at 4–6 months of age – who later go on to develop autism at three years (when behavioural atypicalities become evident) – it can be seen, using this paradigm, that the auditory non-socially selective response is of a greater magnitude than in low risk age matched infants (Lloyd-Fox, Johnson, personal communication). Further, the magnitude and latency of the response appears to differ for the visual social stimuli in high versus low risk infants (Lloyd-Fox et al., 2013). This research suggests that atypical brain responses may be measurable long before behavioural symptoms become apparent (which typically manifest between 2 and 3 years of age). These developmental haemodynamic response curve markers of timing and magnitude are ideal candidates to follow in future research in infants at risk (Aslin, 2012, Vanderwert and Nelson, 2014). The current findings are however limited to the study paradigm employed and may not be directly applicable to the impact of environmental early life risk factors. As longitudinal prospective research continues in this field, increased sample sizes and age points, and fNIRS paradigms that investigate responses across multiple underlying core constructs, rather than just the social domain, will allow us to interrogate antecedent biomarkers of compromised development (such as poverty and under nutrition) in greater depth. Furthermore, the collection of this data from as early in life as possible should allow one to identify how different factors (such as maternal under-nutrition, family poverty, or infant under-nutrition, caregiving practices) compound, or compensate for, risk for compromised development. Importantly, neuroimaging measures such as fNIRS allows us to identify markers of atypical function from a far earlier age (i.e. from birth) than behavioural assessments are typically able to (from 2 to 3 years onwards). Larger scale prospective longitudinal studies could allow us to identify individuals at greatest risk, target additional family support to those families with the greatest need and target interventions from an early age before critical developmental milestones have been affected. The long-term aim of our research is to establish fNIRS as a universal assessment tool for the investigation of the impact of adversity on cognitive development in infants irrespective of where those infants might have been born.

## Conflict of interest

None.
